# Triphenylphosphine: MAK Value Documentation, addendum – Translation of the German version from 2022

**DOI:** 10.34865/mb60335e9_4ad

**Published:** 2024-12-23

**Authors:** Andrea Hartwig, MAK Commission

**Affiliations:** 1 Institute of Applied Biosciences. Department of Food Chemistry and Toxicology. Karlsruhe Institute of Technology (KIT) Adenauerring 20a, Building 50.41 76131 Karlsruhe Germany; 2 Permanent Senate Commission for the Investigation of Health Hazards of Chemical Compounds in the Work Area. Deutsche Forschungsgemeinschaft, Kennedyallee 40, 53175 Bonn, Germany. Further information: Permanent Senate Commission for the Investigation of Health Hazards of Chemical Compounds in the Work Area | DFG

**Keywords:** triphenylphosphine, neurotoxicity, skin absorption, sensitization, toxicity, developmental toxicity, developmental neurotoxicity, genotoxicity, Luft, air

## Abstract

The German Commission for the Investigation of Health Hazards of Chemical Compounds in the Work Area (MAK Commission) has re-evaluated the occupational exposure limit value (maximum concentration at the workplace, MAK value), the pregnancy risk group, and the data for sensitization, absorption through the skin and germ cell mutagenicity of triphenylphosphine [603-35-0]. Relevant studies were identified from a literature search and also unpublished study reports were used. The critical effect is neurotoxicity which was observed in a 4-week study in dogs at a respirable aerosol concentration of 30 mg triphenylphosphine in xylene/m^3^ with a NOAEC of 10 mg/m^3^. Based on this and taking into account the increased respiratory volume at the workplace, the MAK value is set at 2 mg/m^3^ as the inhalable fraction (I). Since a systemic effect is critical, Peak Limitation Category II is retained. The default excursion factor of 2 has been confirmed because the half-life is still not known. There is an adequate margin between the NOAEL for developmental toxicity and the MAK value. However, triphenylphosphine is a neurotoxin and data on developmental neurotoxicity are lacking. Therefore, triphenylphosphine is assigned to Pregnancy Risk Group D. Triphenylphosphine is not mutagenic in bacteria and neither clastogenic in vitro nor in vivo. Skin contact is expected to lead to a relatively minor contribution to systemic toxicity. Triphenylphosphine can cause sensitization of the skin in animals and is therefore designated with “Sh”.

**Table:** 

**MAK value (2021)**	**2 mg/m^3^ I (inhalable fraction)**
**Peak limitation (2007)**	**Category II, excursion factor 2**
	
**Absorption through the skin**	**–**
**Sensitization (2007)**	**Sh**
**Carcinogenicity**	**–**
**Prenatal toxicity (2021)**	**Pregnancy Risk Group D**
**Germ cell mutagenicity**	**–**
	
**BAT value**	**–**
	
CAS number	603-35-0
Vapour pressure at 20 °C	1.2 × 10^–6^ hPa (calculated; ECHA 2019)
log K_OW_	5.69 (OECD 2006)
Soluble at 25 °C	0.09 mg/l water (OECD 2006)

For triphenylphosphine, documentation is available from 2008 (Greim 2008, available in German only).

In 2016, the Commission began using a revised approach for assessing substances with a MAK value based on systemic effects and derived from inhalation studies in animals or studies with volunteers at rest; this new approach takes into account that the respiratory volume at the workplace is higher than under the experimental conditions. This applies only to gases and vapours with a blood:air partition coefficient > 5 and to aerosols (see DFG 2021, Section I b and I c). Triphenylphosphine is a solid and is present as an aerosol, therefore the increased respiratory volume has to be taken into account. This addendum evaluates whether the MAK value and the pregnancy risk group for triphenylphosphine need to be re-assessed as a result of the higher respiratory volume at the workplace.

Studies or publications investigating other end points that were conducted or published since the 2008 documentation (Greim 2008) are also described. Cited unpublished toxicological studies from companies have been made available to the Commission.

## Mechanism of Action

The critical effect after inhalation and oral exposure to triphenylphosphine is neurotoxicity; the mode of action remains unclear. An effect similar to that known from organophosphates, in particular the inhibition of the neurotoxic esterase in the brain, was excluded as the cause in studies in ferrets after subcutaneous injection of 250 or 500 mg triphenylphosphine/kg body weight (in arachis oil/ethyl ether) and examination after 24 hours. The activity of acetylcholinesterase likewise remained unchanged (Greim 2008).

In beagle dogs, the degeneration of axons in the spinal cord was found at and above oral doses of 5 mg/kg body weight and day or inhalation exposure to 30 mg/m^3^ for 4 weeks; this was regarded as a neuropathological correlate for the ataxia that occurred. In the European ferret, a single subcutaneous dose of ≥ 250 mg/kg body weight likewise caused ataxia and, after 6 days, paralysis of the forelimbs and hind limbs. The brainstem, cerebellum, midbrain and forebrain of the animals displayed extensive degeneration of the axons (Greim 2008; OECD 2006).

## Toxicokinetics and Metabolism

There are still no specific studies of the toxicokinetics and metabolism of triphenylphosphine. Systemic effects after inhalation or oral exposure show that triphenylphosphine is at least partially absorbed; this is dependent, however, to a large extent on the solvent and the mode of application. The absorption of the substance after oral administration is significantly improved by lipophilic vehicles. The absorption and systemic availability of triphenylphosphine after inhalation are likewise dependent on the use of a solvent that can act as a vehicle. Particle size is then also likely to be important for the extent of absorption (Greim 2008).

Data for a commercial product manufactured as a dust show that it does not reach the thoracic level due to the large aerodynamic diameter (90% < 1541 μm; 50% < 854 μm; 10% < 111 μm; 0.02% < 10 μm) (ECHA 2019). It is unclear whether the substance is or can be manufactured in respirable diameters.

Earlier model calculations according to Guy and Potts (1993) and Wilschut et al. (1995) yielded very low fluxes of 1.4 × 10^–5^ and 8.3 × 10^–5^ mg/cm^2^ and hour, respectively, which would lead to the absorption of 28 and 166 µg after the exposure of both hands and forearms (an area of about 2000 cm^2^) for 1 hour (see Greim 2008). Using the IH SkinPerm model (Tibaldi et al. 2014), the calculated amount of triphenylphosphine absorbed in this scenario is similarly only 35.2 µg, whereas the model of Fiserova-Bergerova et al. (1990) yields an absorbed amount of 2.9 mg.

## Effects in Humans

There are no data available.

## Animal Experiments and in vitro Studies

### Acute toxicity

In rats, the LC_50_ after inhalation exposure for 4 hours in whole-animal chambers was 12 500 mg/m^3^. Respiratory irritation was observed as a clinical sign of toxicity. The critical effect after acute oral administration of triphenylphosphine in rodents, chickens and dogs is neurotoxicity. The oral LD_50_ in rats ranged from 700 mg/kg body weight (in olive oil) to over 6400 mg/kg body weight (as an aqueous suspension), depending on the solvent used. In mice, an oral LD_50_ of 1000 mg/kg body weight (in olive oil) was obtained. The dermal LD_50_ is above 2500 mg/kg body weight in rats and above 4000 mg/kg body weight in rabbits. The animals were given a 50% alcoholic suspension of triphenylphosphine and did not display any signs of systemic toxicity or skin irritation (Greim 2008).

### Subacute, subchronic and chronic toxicity

New data from animal studies after repeated administration are not available. The studies that are decisive for the derivation of the MAK value are presented below. The description of further studies can be found in the documentation of 2008 (Greim 2008).

#### Inhalation

Groups of 6 male CD rats were whole-body exposed to a measured triphenylphosphine concentration of 0 or 2400 mg/m^3^ for 4 hours per day, on 5 days per week, for 12 days. For exposure, heated liquid triphenylphosphine was nebulized with dry nitrogen and enriched with oxygen. To determine the analytical concentrations, samples of the exposure atmosphere were collected in an evacuated vessel, taken up with ethanol and determined photometrically after dilution. Three animals each from the control and treatment groups were sacrificed and examined immediately after the last exposure and the remaining animals 14 days after the end of exposure. The treated animals displayed salivation and lacrimation, laboured breathing and reddened ears during exposure, indicating irritation. During the second week of exposure, brownish discoloration of the fur was seen, which disappeared immediately after the last exposure. Necropsy and histopathology, including of the brain, did not reveal any treatment-related findings. There were no unusual findings in the lungs and trachea (Waritz and Brown 1975). The particle size was not specified. The larynx, where aerosols often settle, was not examined histopathologically. Clinical neurotoxic effects like those found in the following study in dogs were not reported.

Neurotoxic effects were induced in beagle dogs in inhalation studies which used xylene as a solvent to improve the solubility of triphenylphosphine. In these studies, 1 male and 1 female animal per treatment group were exposed to an aerosol of a xylene/triphenylphosphine solution at concentrations of 0.5, 3.2, 9.7 or 28 mg triphenylphosphine/m^3^ (nominally 0.5, 3, 10 and 30 mg/m^3^, particle size ≤ 0.5 μm; whole-body exposure; xylene concentration ≤ 150 ml/m^3^) for 6 hours daily, on 5 days per week, for 4 weeks. One male and 1 female dog per group were exposed to 0.3, 3.6 or 24 mg/m^3^ for 6 hours per day on 2 consecutive days. Histopathological examination was carried out 4 weeks after the end of exposure. Only the brain, right and left sciatic nerve and lumbar and cervical spinal cord were examined histopathologically. The male animals of the high concentration group (1 animal after 2-day and 1 after 4-week exposure) exhibited clinical signs of neurotoxicity (for example, coordination disorders and weakness of the hind legs). Histologically, vacuolar degeneration of the axons in the cervical and lumbar medulla was found in the high concentration group after 4 weeks but not after 2 days of exposure, and not in the sciatic nerve. In the lower concentration groups, slight neurological effects were observed sporadically, but without clear histological findings and without concentration dependence, so that these effects were probably not exposure-related. Based on the clinical signs of ataxia and degenerative lesions in the spinal cord, the NOAEC (no observed adverse effect concentration) of this study was 10 mg/m^3^ after 4 weeks. The control animals exposed only to the CNS depressant xylene did not display any clinical signs of neurotoxicity or histopathological abnormalities (M & T Chemicals Inc 1982 a, 1983). Sacrifice and histopathological examination of the animals which had only been exposed for 2 days took place 4 weeks later. Since the effects may have been reversible, the negative result of this examination is not meaningful. However, the animals exhibited clinical signs of neurotoxicity.

**Conclusions**: According to the available data, a prerequisite for neurotoxic effects after inhalation seems to be the dissolution of triphenylphosphine in a lipophilic vehicle and absorption in droplet form. Sufficient bioavailability is apparently not achieved in dust form (Greim 2008).

The lowest NOAEC is that obtained in a 4-week inhalation study in beagle dogs of 10 mg/m^3^.

#### Oral administration

In a subchronic toxicity study according to OECD Test Guideline 408 with neurotoxicity examinations, groups of 10 male and 10 female Wistar rats were given triphenylphosphine doses of 0, 6, 60 or 120 mg/kg body weight and day (as an aqueous preparation) daily for 90 days by gavage. At 60 and 120 mg/kg body weight and day, centrilobular hypertrophy of the hepatocytes was observed and in the females the absolute (middle dose group: +16%, high dose group: +31%) and relative (+11% and +30%) liver weights were increased and the prothrombin time in plasma was decreased. At 120 mg/kg body weight and day, the liver weights were increased with statistical significance also in the males (absolute: +31%, relative: +30%) and the kidney weights in the females (absolute: +15%, relative: +13%). Clinico-chemical changes (for example, an increase in calcium, total protein and triglycerides) were observed at this dose, which together with the increase in organ weights indicate enzyme induction. In the neuropathological examination, axonal degeneration was found in the proximal sciatic nerve in 1 of 5 examined female animals of the 120 mg/kg dose group and axonal degeneration was seen in the sural nerve of another female. No statistically significant differences were observed between animals in the control and treatment groups in a functional observational battery (FOB) and motor activity assessments. The NOAEL (no observed adverse effect level) was 6 mg/kg body weight and day (BASF AG 2002).

In a 4-week study with gavage administration of 0, 1, 5, 10 or 20 mg triphenylphosphine/kg body weight and day, on 5 days per week, to 1 male and 1 female beagle dog per dose, neurological impairments such as failure of the patellar reflex were observed in male animals even at the dose of 5 mg/kg body weight and day and above. Corn oil was used as the solvent. Histopathological examination, limited to the brain, the right and left sciatic nerve and the lumbar and cervical spinal cord, revealed vacuolar degeneration of the axons in the cervical and lumbar spinal cord in male and female animals at 10 mg/kg body weight and day and above, but not in the sciatic nerve. The NOAEL in this study was 1 mg/kg body weight and day. In addition, 1 male and 1 female dog per dose received 1, 5 or 20 mg/kg body weight and day for 2 days only, with a 4-week treatment-free recovery period. The NOAEL in this study was 20 mg/kg body weight and day (Greim 2008; M & T Chemicals Inc 1982 b, 1983).

**Conclusions**: Neurological effects were seen also after repeated oral administration, depending on the form of preparation. As a prerequisite for neurotoxicity, triphenylphosphine must be absorbable and bioavailable in the nervous system. When administered orally, this is apparently better achieved with an oily preparation than with an aqueous one, as illustrated by the rat and dog studies described in this section. However, a difference in species sensitivity cannot be ruled out either. The lowest NOAEL was obtained in a 4-week gavage study in beagle dogs and was given as 1 mg/kg body weight and day with corn oil as the solvent.

### Local effects on skin and mucous membranes

Triphenylphosphine causes slight irritation of the skin and eyes (Greim 2008).

### Allergenic effects

There are no new data available. In the guinea pig maximization test already described in the documentation of 2008, triphenylphosphine was found to have low sensitization potency (Greim 2008).

### Reproductive and developmental toxicity

#### Fertility

Specific studies of the effect of triphenylphosphine on fertility are still not available. As described in the documentation of 2008, in a 90-day gavage study in rats, neither weight changes nor unusual histopathological findings in the reproductive organs were observed up to the highest dose tested of 120 mg triphenylphosphine in aqueous preparation per kg body weight and day. A slightly decreased sperm count in the high dose group in comparison with that of historical controls was not regarded as relevant (Greim 2008).

#### Developmental toxicity

In the documentation of 2008, a developmental toxicity study in Wistar rats carried out according to OECD Test Guideline 414 was described. The dams were given gavage doses of 0, 10, 30 or 90 mg triphenylphosphine/kg body weight and day (in aqueous suspension with 0.5% carboxymethylcellulose) from gestation days 6 to 19. In the high dose group, variations in the form of ossification delays occurred in the foetuses, which were regarded as substance-related by the Commission. At this dose, food intake and body weight gains were reduced from days 6 to 8 of gestation in the dams. Therefore, the NOAEL for developmental toxicity and maternal toxicity was established to be 30 mg/kg body weight and day (Greim 2008).

### Genotoxicity

#### In vitro

Triphenylphosphine was not mutagenic in bacteria (Greim 2008). Treatment of human gingival fibroblasts with 0.24, 0.8 or 2.4 mM triphenylphosphine (= 1/10 EC_50_, 1/3 EC_50_ and EC_50_, respectively) for 6 hours in an XTT cell viability assay resulted in increased phosphorylation of histone H2AX (γ-H2AX) with a maximum at 2.4 mM (9 ± 0.66 foci/cell, control: 0.65 ± 0.04). At this concentration, 6.8 ± 0.2 (8.5%) of 80 counted cells contained more than 40 foci (multi-foci cells), 0.52 ± 0.26 were apoptotic, and 1.32 ± 0.27 were necrotic (Shehata et al. 2013). The phosphorylation of H2AX is an early event in DNA repair. Usually DNA double-strand breaks are the trigger, but also single-strand breaks, e.g. triggered by UV radiation or oxidative stress (Mishima 2017) and DNA damage during proliferation or apoptosis can cause phosphorylation of H2AX. The increased γ-H2AX antibody binding is therefore not exclusively evidence of induced double-strand breaks, but of DNA damage in general. Since Shehata et al. (2013) counted foci in cells, the data were not confounded by apoptosis, which was also observed; apoptosis leads to uniform staining of the nucleus and not to foci (Solier and Pommier 2009). To prove beyond doubt that these foci are DNA double-strand breaks, the co-localization of a second protein (for example, 53BP1) must be demonstrated (Rothkamm et al. 2015). Since this was not carried out in the present case, the increase in not further specified DNA damage was determined here by the quantification of γ-H2AX.

In a micronucleus test without the addition of metabolic activation, no clastogenicity was found in CHL cells (a cell line derived from Chinese hamster lung) up to the cytotoxic concentration. An assay with the addition of metabolic activation was not performed and the results of the different concentrations are not documented in the original publication (see Greim 2008).

An HPRT gene mutation test with CHO cells (a cell line from Chinese hamster ovary) according to OECD test guideline 476 yielded negative results. After 4 hours of treatment, concentrations between 1.56 and 12.5 μg/ml or, in a second experiment, between 1.25 and 10 μg/ml without the addition of a metabolic activation system, and 2.5 to 40 μg/ml or, in the second experiment, 3.13 to 50 μg/ml in the presence of a metabolic activation system were evaluated. Higher concentrations were cytotoxic. The purity was 99.71%. Tetrahydrofuran was used as the solvent. The positive controls 7,12-dimethylbenzanthracene and ethyl methanesulfonate produced the expected results, indicating a functioning test system (BASF SE 2014).

#### In vivo

There are no new data available. A bone marrow micronucleus test in mice with intraperitoneal administration, with limited documentation, yielded negative results. The highest dose used was 80% of the LD_50_ of triphenylphosphine in corn oil (no other details) (Greim 2008).

#### Summary

The available in vitro and in vivo studies, some of which with only limited documentation, give no indication that triphenylphosphine has genotoxic effects, except for the induction of not further specified DNA damage in gingival fibroblasts observed in an indicator test in vitro.

## Manifesto (MAK value/classification)

Critical effects are neurotoxicity and skin sensitization.

**MAK value. **Since the documentation from 2008 (Greim 2008) there are no new data that could be used to derive a MAK value. The MAK value was established on the basis of the NOAEC (no observed adverse effect concentration) of 10 mg/m^3^ from the 4-week inhalation study in dogs (M & T Chemicals Inc 1982 a, 1983). Clinical signs of neurotoxicity occurred in dogs after exposure to 24 mg/m^3^ for 2 days and did not increase after 4 weeks of exposure to 28 mg/m^3^. For this reason, the effects are not assumed to increase with chronic exposure. The results of the histopathological examination cannot be used to assess whether an increase in effects is possible because the animals of the 2-day study were not examined until 4 weeks after the end of exposure and possible effects might already have been reversible. The extrapolation from the LOAEC (lowest observed adverse effect concentration) of 24 mg/m^3^ from the 2-day study (1 : 3) leads to a NAEC (no adverse effect concentration) of 8 mg/m^3^ and corresponds approximately to the NOAEC after 4 weeks of exposure. Since the NOAEC of 10 mg/m^3^ from the 4-week study is derived from animal studies (1 : 2), the MAK value, taking into consideration the increased respiratory volume (1 : 2) and the preferred value approach, is 2 mg/m^3^ for the inhalable fraction.

A NOAEL (no observed adverse effect level) of 1 mg/kg body weight and day (dissolved in corn oil) was obtained from the 4-week oral study in dogs. The LOAEL (lowest observed adverse effect level) is 5 mg/kg body weight and day. The following toxicokinetic data are taken into consideration for the extrapolation of this NOAEL to a concentration in workplace air: the species-specific correction value (1 : 1.4) for the dog, the assumed oral absorption (100%), the body weight (70 kg) and respiratory volume (10 m^3^) of the person, and the assumed 100% absorption by inhalation. The concentration calculated from this is 5 mg/m^3^. Since this value is derived from a NOAEL from experimental studies in animals (1 : 2), a limit value in air of 2 mg/m^3^ would likewise result, taking into account the preferred value approach.

In an oral 90-day study in the rat, a NOAEL of 6 mg/kg body weight and day was obtained for triphenylphosphine in an aqueous solution (BASF AG 2002). At and above 60 mg/kg body weight and day, there was an increase in liver weights and a decrease in prothrombin time; neurotoxic effects were observed only at 120 mg/kg body weight and day. The oral studies in rats suggest poorer absorption of the substance from aqueous solution compared with the triphenylphosphine administered in corn oil in the studies in dogs. Absorption is also much lower after inhalation of powdered triphenylphosphine compared with the substance prepared in xylene solution. The inhalable fraction is not absorbed after inhalation and deposition in the respiratory tract, but is probably swallowed after mucociliary clearance and then enters the gastrointestinal tract. The low oral absorption therefore plays a role also after inhalation. Absorption after oral and inhalation exposure can therefore be assumed to be largely similar and need not be included in the following calculation. The following toxicokinetic data are taken into consideration for the extrapolation of the NOAEL of 6 mg/kg body weight and day to a concentration in workplace air: the daily exposure of the animals in comparison with the 5 days per week exposure at the workplace (7 : 5), the corresponding species-specific correction value for the rat (1 : 4), the body weight (70 kg) and respiratory volume (10 m^3^) of the person. The concentration calculated from this is 14.7 mg/m^3^. As this value is derived from a NOAEL from experimental studies in animals (1 : 2), a MAK value of 5 mg/m^3^ for the inhalable fraction can be derived using the preferred value approach. This value is higher than the MAK value obtained from the data in dogs. The NOAEC of 10 mg/m^3^ from the dog study thus represents a worst case scenario and continues to serve as the basis for the MAK value of 2 mg/m^3^ I. Three studies in two species with oral and inhalation exposure thus result in very similar MAK values.

The MAK value for triphenylphosphine has been lowered to 2 mg/m^3^ I.

The derivation of a MAK value for the respirable fraction for triphenylphosphine is not considered necessary, since less than 0.02% of a commercially available product has an aerodynamic diameter of less than 10 µm (ECHA 2019). It is unclear whether the substance is or can be produced in respirable diameters.

**Peak limitation. **As the MAK value for triphenylphosphine is derived from systemic effects, the assignment to Peak Limitation Category II remains valid. The default excursion factor of 2 for substances with systemic effects has been confirmed because the half-life is still not known.

**Prenatal toxicity. **There are no new data for the developmental toxicity of triphenylphosphine. The developmental toxicity data in Wistar rats indicate ossification delays only at the maternally toxic dose of 90 mg/kg body weight and day. The NOAEL for developmental and maternal toxicity is 30 mg/kg body weight and day. For the extrapolation of this NOAEL, the same toxicokinetic parameters are applied as described in the Section “MAK value” but without the conversion to 5 days per week exposure. This results in a concentration in air of 52.5 mg/m^3^, which corresponds to a 26-fold margin to the MAK value of 2 mg/m^3^. The margin to the MAK value is therefore sufficiently large so that, as regards developmental toxicity, triphenylphosphine could continue to be assigned to Pregnancy Risk Group C. However, the MAK value for triphenylphosphine is derived from neurotoxic effects, so that developmental neurotoxicity must be considered.

Since 2016, the Commission considers a statement on developmental neurotoxicity in the foetus to be necessary for substances whose MAK value was derived from neurotoxic effects. Studies of the end points neurotoxicity or behavioural toxicity in offspring exposed in utero are not available for triphenylphosphine. There are no data for toxicokinetics, metabolism or the mechanism of action that would allow a statement on whether foetuses react less sensitively to triphenylphosphine than adult animals. Thus, a statement on the developmental neurotoxicity of triphenylphosphine in the foetus is not possible.

Triphenylphosphine has therefore been reclassified: from Pregnancy Risk Group C to Pregnancy Risk Group D.

**Germ cell mutagenicity. **Triphenylphosphine is not mutagenic in bacteria and mammalian cells in vitro. In an indicator test in vitro, not further specified DNA damage was induced in gingival fibroblasts. Triphenylphosphine is not clastogenic in mammalian cells. An incompletely documented micronucleus test in mouse bone marrow with intraperitoneal administration yielded negative results. Tests with germ cells are not available. In view of the data, triphenylphosphine has not been classified in one of the categories for germ cell mutagens.

**Absorption through the skin. **There are still no data available for the absorption of triphenylphosphine through the skin. From model calculations, a maximum absorbed amount of 2.9 mg through 2000 cm^2^ of skin was calculated for an exposure duration of 1 hour. Assuming a total respiratory volume of 10 m^3^ and quantitative absorption, an 8-hour exposure at the level of the MAK value would result in an absorbed amount by inhalation of 20 mg of triphenylphosphine. In comparison, the amount dermally absorbed is low. Therefore, triphenylphosphine has not been designated with an “H” (for substances which can be absorbed through the skin in toxicologically relevant amounts).

**Sensitization. **There are still no data for the sensitizing effects of triphenylphosphine on the skin in humans and no new animal studies. Due to the positive result of the maximization test in guinea pigs already assessed in the documentation of 2008 (Greim 2008), triphenylphosphine remains designated with “Sh” (for substances which cause sensitization of the skin). There are no data for respiratory sensitization, so that triphenylphosphine has not been designated with “Sa” (for substances which cause sensitization of the airways).
